# Dietary Choline and Betaine Are Not Associated With the Risk of Type 2 Diabetes. A Systematic Review and Meta‐Analysis of Observational Studies

**DOI:** 10.1155/jdr/9980163

**Published:** 2026-01-01

**Authors:** Elham Sharifi-Zahabi, Sobhan Mohammadi, Fatemeh Hajizadeh-Sharafabad, Nooshin Nasiri, Fatemeh Sadeghi, Amir Saber

**Affiliations:** ^1^ Student Research Committee, Kermanshah University of Medical Sciences, Kermanshah, Iran, kums.ac.ir; ^2^ Department of Clinical Nutrition, School of Nutritional Sciences and Dietetics, Tehran University of Medical Sciences, Tehran, Iran, tums.ac.ir; ^3^ Patient Safety Research Center, Clinical Research Institute, Urmia University of Medical Sciences, Urmia, Iran, umsu.ac.ir; ^4^ Exercise Physiology Department, Islamic Azad University Central Tehran Branch, Tehran, Iran, iauctb.ac.ir; ^5^ Department of Nutritional Sciences, School of Nutritional Sciences and Food Technology, Kermanshah University of Medical Sciences, Kermanshah, Iran, kums.ac.ir

**Keywords:** betaine, choline, meta-analysis, Type 2 diabetes

## Abstract

**Background:**

The link between dietary choline/betaine and Type 2 diabetes (T2D) has been shown in several studies. However, the findings are controversial. This meta‐analysis evaluated the connection between dietary choline/betaine and the incidence of T2D.

**Methods:**

A thorough and organized search was conducted through PubMed, Scopus, and Web of Science up to September 2024. All reported effect sizes and their 95% CIs for risk of T2D were used to estimate log RRs with their standard errors (SEs). The overall combined effect size was obtained via a random effects model. The variability among the studies was examined using Cochrane′s *Q* test and the *I*‐squared statistic.

**Results:**

A total of five studies, including 76,678 subjects, provided data on the relationship between dietary choline and T2D risk. Compared with the lowest category of dietary choline, the combined RR for the diabetes incidence was 1.15 (95% CI: 1.00, 1.33; *p* = 0.058; *I* ^2^ = 63.1*%*) for the highest category of dietary choline. The pooled analysis of three studies, with 65,725 subjects, indicated no significant link between dietary betaine and T2D incidence. Compared with the lowest category of dietary betaine, the combined RR for the diabetes incidence was 0.99 (95% CI: 0.90, 1.10; *p* = 0.871; *I* ^2^ = 58.1*%*). The certainty of the evidence was rated very low for choline intake and T2D incidence as well as betaine intake and T2D incidence, with a downgrade for risk of bias, inconsistency, imprecision, and indirectness.

**Conclusion:**

The findings of this study do not advocate the role of dietary choline/betaine in T2D incidence. Due to the limited number of primary studies and high heterogeneities among them, more rigorously designed prospective studies are required to confirm our results.

## 1. Introduction

Type 2 diabetes (T2D) is identified as a serious and chronic condition marked by impaired blood glucose levels resulting from insufficient insulin production or an inability of the body to utilize insulin properly [1]. Diabetes poses a significant financial strain on global healthcare systems [2]. Estimates reveal that 643 million more people worldwide will have diabetes by 2030 [3]. Although the precise causes of T2D remain unclear, obesity, increasing age, race, and family history are strongly linked to the development of the disease [4]. Dietary intake also plays a vital role in the advancement of cardiometabolic disorders like diabetes mellitus [5]. The connection between dietary factors and the risk of T2D is well recognized [5, 6]. However, our knowledge about the potential association between the risk of T2D and particular dietary nutrients like choline and betaine is insufficient. Choline is crucial in human metabolism since it serves as an important supplier of methyl groups, an antecedent for the neurotransmitter acetylcholine, and a constituent of cell membranes [7]. Foods supplied in choline and betaine (oxidized from choline, which helps turn homocysteine to methionine), such as red meat, eggs, and whole grains, can have both favorable and adverse effects on chronic diseases [7]. Regarding the function of choline and betaine in the one‐carbon cycle, growing interest exists in how dietary choline and betaine found in various foods are related to disease risk in humans due to the possible epigenetic effects [7, 8].

Data regarding the correlation between dietary choline/betaine and the likelihood of developing T2D in humans is limited, and the results have been inconsistent [8, 9]. Studies indicate that higher dietary choline intake is linked to attenuated levels of inflammatory factors like C‐reactive protein and interleukin‐6, so choline has the potential to improve inflammation [10]. However, choline is a vital dietary source for gut bacteria that produce trimethylamine, which is then transformed by the liver to trimethylamine N‐oxide (TMAO) [11]. Studies revealed a link between high levels of TMAO in the bloodstream and increased risk of metabolic disorders such as T2D and cardiovascular disease (CVD) [12, 13]. Furthermore, a recent cohort study pointed out that subjects with higher dietary consumption of phosphatidylcholine are more likely to have T2D [9]. In the Lever et al. study, individuals with T2D often have reduced levels of betaine in their blood compared with those without this condition [14, 15]. Another prospective cohort study indicated a lower risk of T2D among men consuming choline [8]. Given the limited and conflicting studies on the association between dietary choline/betaine and the risk of T2D, the purpose of this study was to conduct a systematic review and meta‐analysis on observational studies that have examined the link between dietary choline/betaine intake and the incidence of T2D.

## 2. Methods

The protocol of the current study was based on the Preferred Reporting Items for Systematic Reviews and Meta‐Analyses (PRISMA) 2020 instructions and was registered in the PROSPERO database. ID: CRD42024588932.

### 2.1. Search Strategy and Criteria For Study Selection

The electronic databases PubMed, Scopus, Web of Science, and Google Scholar were rigorously investigated up to September 2024. The medical subject headings (MeSH) and related keywords were applied in searching the literature. The query terms relevant to “choline,” “betaine,” and “diabetes,” along with their combinations, were applied to find the related publications Table S1. We also checked the references section of eligible studies to find additional relevant publications. No limitation on date was applied while searching literature. We applied the PICOS scheme as follows: population: adults aged ≥ 18 years; intervention: dietary choline/betaine; comparator: highest intake vs. lowest intake; Outcome: T2D; Study design: observational studies that provide odds ratios (ORs), risk ratios (RRs), or hazard ratios (HRs) coupled with their 95% confidence intervals for the relationship between dietary choline or betaine and T2D. The letters, comments, reviews, meta‐analyses, ecological studies, and case reports were omitted. These exclusions were considered for studies that enrolled pregnant women, children, or adolescents. Two researchers approved the excluded publications. Any discrepancies were resolved through discussion with the principal author.

### 2.2. Extraction of Data

Employing a data extraction checklist, the required data were chosen and extracted by one author and checked for accuracy by another researcher. Qualitative data include the first author, country, publication date, study design, population characteristics, follow‐up duration, exposure, age, related effect sizes, number of total participants, number of cases, and any adjustment made for confounders. In the studies where various effect sizes were presented for T2D risk, the effect sizes that were adjusted for the higher number of confounders were selected.

### 2.3. Risk of Bias Assessment

The studies were evaluated using the Risk Of Bias In using the Non‐randomized Studies of Exposure effects (ROBINS‐E) checklist [16]. This checklist consists of seven essential components assessing bias related to (1) confounding variables, (2) participant selection, (3) exposure evaluation, (4) misclassification during follow‐up, (5) missing data, (6) outcome measurement, and (7) selective reporting of results.

### 2.4. Grading the Evidence

The certainty of the meta evidence was assessed using the Grading of Recommendations Assessment, Development, and Evaluation (GRADE) method, which is a framework for evaluating the reliability of the evidence [17]. This tool categorizes observational studies as low quality, applying downgrades for study limitations, inconsistency, indirectness, imprecision, and publication bias, while providing upgrades for large effect sizes, dose‐response relationships, and attenuation by plausible confounding.

### 2.5. Statistical Analysis

In this meta‐analysis, all reported effect sizes (along with their 95% CIs) for the association between dietary choline/betaine and T2D were considered relative risks (RRs). The combined effect size was determined by employing a random effects model that considers the variability between studies. The assessment of interstudy heterogeneity was based on Cochrane′s *Q* test and *I*‐squared (*I* ^2^). Subgroup analysis based on the evaluation method of choline intake was conducted to investigate potential sources of heterogeneity among studies using the random effects model.

To determine whether the combined effect size could be affected by a particular study, a sensitivity analysis was conducted by systematically deleting each study from the analysis one by one at a time including the cross‐sectional study by Zhou et al. [18]. In addition, publication bias was assessed using a visual inspection of funnel plots and statistically tested by Egger′s and Begg′s regression tests [19, 20]. All statistical analyses were done by Stata, Version 14 (StataCorp). Values of < 0.05 were considered statistically significant.

## 3. Results

### 3.1. Literature Search

**Figure 1:**
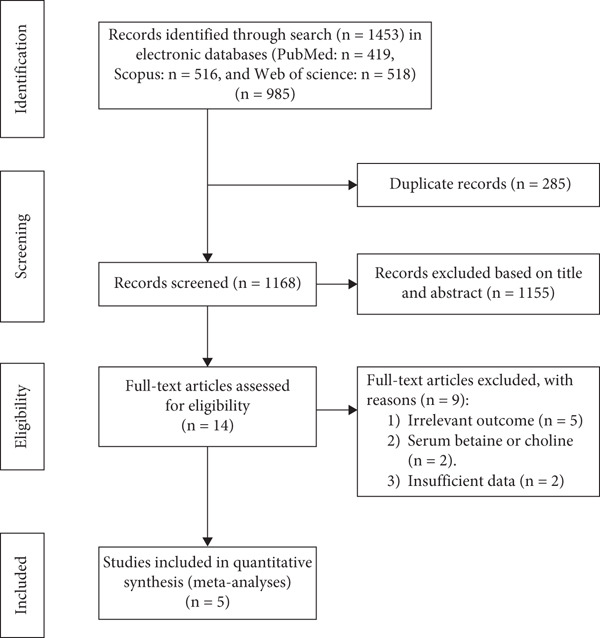
Processes of studies selection.

The summarized process of the study selection is illustrated in Figure 1. Following our primary systematic search in online databases, 1453 studies were determined. Excluding duplicates and irrelevant studies led to the remaining 14 possibly suitable articles for additional assessment. After reviewing the full texts, an additional nine studies were removed because of the reasons mentioned as follows: five studies presented unrelated outcomes [21–25], two assessed the serum choline [26] or betaine [27] in relation to T2D, and one study did not provide sufficient data [9, 28]. Finally, five studies with a total of 76,678 participants and 8929 cases of T2D were included in this meta‐analysis [8, 18, 29–31].

### 3.2. Study Characteristics

Table 1 displays the features of the studies included in this meta‐analysis. Three of the included studies originated from the USA [18, 29, 32], one was from Iran [31], and the other from Finland [8]. All the studies were published between 2020 and 2023. Except for the study by Zhou et al. [18], which had a cross‐sectional design, the other studies were prospective cohort studies. Although all the included studies assessed dietary choline as an exposure, three of them also examined dietary betaine. A total of 76,678 subjects aged 40.2–65.7 years were included in the meta‐analysis, from which 8929 cases of T2D were diagnosed. The follow‐up duration ranged between 6.63 and 19.3 years. While three studies included both males and females, the studies by Greenberg et al. [32] and Virtanen et al. [8] were conducted exclusively on females and males, respectively. All studies adjusted for sex, age, BMI, smoking, and energy intake. Some others also controlled for micronutrients, family history of diabetes, dietary fats and protein, alcohol consumption, race, and physical activity.

**Table 1:** Main characteristics of studies examining the association between dietary choline and betaine and the risk of Type 2 diabetes.

**Author/year**	**Country**	**Study design**	**Exposure**	**Patients characteristic**	**Participants/cases**	**Follow up duration**	**Age (y) (mean)**	**Sex**	**Adjustment**
Hosseini‐Esfahani et al. [31]	Iran	Cohort	Choline/betaine	Tehranian adults.	6022/528	6.0.63	40.2	M/F	Sex and age adjustment, education levels, smoking, BMI, physical activity, family history of T2D, energy intake, TG/HDL ratio, fiber gram per 1000 kcal/day, polyunsaturated fatty acid, dairy, meats, egg, vitamin B12, folate, and coffee plus.

Greenberg et al. [32]	USA	Cohort	Choline/Betaine	Postmenopausal women	46263/5480	13.3	65.7	F	Age and self‐reported race/ethnicity, BMI, 3‐y BMI change, the revised alternative healthy eating index,. energy intake, physical activity, smoking status, alcohol intake in drinks; educational level, and coffee consumption, total cholesterol and total betaine.

Zhou et al. [18]	USA	Cross‐sectional	Choline	Individuals from NHANSE	8621/1093	—	50.4	M/F	Age, sex, ethnicity, education levels, family income levels, smoking status, drinking status, passive sedentary hours, body mass index, C‐reactive protein, dietary energy, saturated fatty acids, polyunsaturated fatty acids, fiber, folate, vitamin B‐6, and vitamin B‐12 intakes.

Dibaba et al. [29]	USA	Cohort	Choline/Betaine	Men and women from four U.S. communities	13440/1396	9	54	M/F	Choline and betaine and further adjusted for age, sex (in overall models), race, log‐BMI, education, smoking, and alcohol, total calorie intake and FBG, magnesium, vitamin B6, vitamin B12, methionine, animal fat, animal protein, and folate intake, FBG, fasting blood glucose.

Virtanen et al. [8]	Finland	Cohort	Choline	Men from eastern Finland	2332/432	19.3	53	M	Age, examination year and energy intake, smoking, body mass index, leisure‐time physical activity, family history of type 2 diabetes, and intakes of alcohol, polyunsaturated fatty acids, and fiber, major dietary sources of choline, and phosphatidylcholine, energy‐adjusted intake of nutrients involved in the choline metabolism, vitamin B12 and folate .

Abbreviations: BMI, body mass index; F, female; HDL, high density lipoprotein; M, male; NHANES, National Health and Nutrition Examination Survey; T2D, Type 2 diabetes; TG, triglyceride.

### 3.3. Meta‐Analysis Results

**Figure 2:**
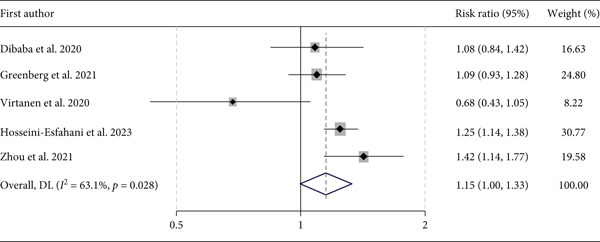
Forest plot of the association between dietary choline and Type 2 diabetes incidence.

A total of five studies, including 76,678 subjects, provided data on the association between dietary intake of choline and T2D risk. Compared with the lowest category of dietary choline, the pooled RR for diabetes incidence was 1.15 (95% CI: 1.00, 1.33, *p* = 0.058) for the highest category of dietary choline. Figure 2. Considering *I* ^2^ index (63.1%) and the Cochrane′s *Q* test (*p* = 0.028), significant heterogeneity was observed across the studies. A sensitivity analysis showed that the study conducted by Virtanen et al. [8] had the largest effect on the results, as after the exclusion of this study from the meta‐analysis, dietary intake of choline had a significant positive association with diabetes risk (RR: 1.20, 95% CI: 1.09, 1.33). Nevertheless, the exclusion of the cross‐sectional study did not change the overall results (RR: 1.09, 95% CI: 0.92, 1.28). Subgroup analysis based on the assessment method of choline intake did not change the result. Although visual examination of the funnel plot showed signs of asymmetry, quantitative analyses with Egger′s did not indicate publication bias (*p* = 0.09).

**Figure 3:**
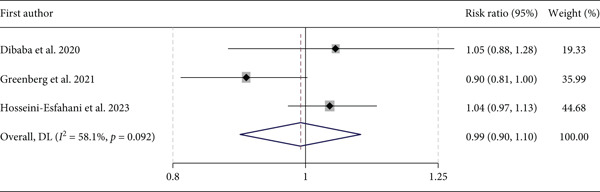
Forest plot of the association between dietary betaine and Type 2 diabetes incidence.

The pooled analysis of three studies, with 65,725 subjects, indicated no significant association between dietary intake of betaine and diabetes incidence. Compared with the lowest category of dietary betaine, the pooled RR for the diabetes incidence was 0.99 (95% CI: 0.90, 1.10, *p* = 0.871). Figure 3. The *I* ^2^ index (58.1%) and Cochrane′s *Q* test (*p* = 0.092) did not reveal significant heterogeneity among the studies. A sensitivity analysis demonstrated that the link between betaine dietary intake and diabetes did not depend on a special study. Visual examination of the funnel plot showed an asymmetry, whereas Egger′s test (*p* = 0.884) did not confirm the presence of publication bias.

### 3.4. Risk of Bias Assessment

Table 2 illustrates the results of the risk of bias assessment for all included studies. Two of the included studies [18, 32] had a serious risk of bias, and the remaining had a moderate risk of bias. Confounding variables were moderately adjusted in three of the included studies [8, 29, 31] (age, sex, BMI, smoking, alcohol, and major dietary source of choline), and the other two studies did not adequately adjust for potential confounders [18, 32]. Two studies had a moderate risk of bias for exposure assessment [8, 18] and misclassification [18, 29] during follow‐up. None of the studies were biased by the participants′ selection, missing data, measurement of the outcome, and selective reporting of the results.

**Table 2:** Risk of bias based on ROBINS‐E judgment for each domain and overall.

**Study**	**Bias due to confounding**	**Bias due to the selection of participants**	**Bias due to exposure assessment**	**Bias due to misclassification during follow-up**	**Bias due to missing data**	**Bias due to measurement of the outcome**	**Bias due to selective reporting of the results**	**Overall judgment**
Dibaba et al. [29]	Moderate	Low	Low	Moderate	Low	Low	Low	Moderate
Greenberg et al. [32]	Serious	Low	Low	Low	Low	Low	Low	Serious
Virtanen et al. [8]	Moderate	Low	Moderate	Low	Low	Low	Low	Moderate
Hosseini‐Esfahani et al. [31]	Moderate	Low	Low	Low	Low	Low	Low	Moderate
Zhou et al. [18]	Serious	Low	Moderate	Moderate	Low	Low	Low	Serious

Abbreviation: ROBINS‐E, Risk Of Bias In Non‐randomized Studies of Exposure effects.

### 3.5. Grading the Evidence

The certainty of the evidence was rated very low for choline intake and T2D incidence as well as betaine intake and T2D incidence, with a downgrade for risk of bias, inconsistency, imprecision, and indirectness (Tables S2 and S3).

## 4. Discussion

In this systematic review and meta‐analysis of observational studies, we aggregated the results of five studies to examine the relationship between dietary choline and betaine and the risk of T2D. While the findings concerning the connection between dietary betaine intake and the risk of T2D were not significant, a positive correlation between dietary choline and diabetes was noted. The direction of the estimate indicates that higher dietary choline intake is associated with an increased risk of diabetes incidence. Specifically, a pooled relative risk of 1.15 suggests that individuals in the highest category of dietary choline intake have a 15% higher risk of developing diabetes compared with those in the lowest category. While the increase is not very large, it is still noteworthy, indicating that dietary choline may play a role in diabetes risk. Additionally, a slightly narrower confidence interval that excludes RR = 1.0 would further suggest a statistically significant association.

According to our current understanding, this study is the first to thoroughly assess the relationship between dietary choline and betaine and the incidence of diabetes. In recent years, there has been increasing interest in the association between dietary choline and betaine and the risk of various diseases. A negative relationship between dietary choline intake and inflammatory factors has been reported by several studies [10, 33]. So, choline can be beneficial in the attenuation of the inflammatory status, a risk factor for CVD and T2D. Although there is a speculation that choline may influence CVDs, several observational studies have shown no clear relationship between dietary consumption of choline and CVD risk [29, 34]. Other research declares that a higher intake of dietary choline may be related to an increased risk of T2D by increasing TMAO [11, 13]. However, in line with our findings, a meta‐analysis of prospective studies revealed no statistically significant relationship between dietary choline or betaine and CVD incidence [35]. Findings from the original studies on the relationship between dietary choline and betaine and the risk of CVDs or T2D are controversial. Zhou et al. [36], in research involving 14,323 participants from the NHANES (National Health and Nutrition Examination Survey), discovered that increased dietary choline consumption was linked to a reduced risk of CVD.

In a cohort study, Karlsson et al. [28] demonstrated a positive association between the higher intake of total choline and an increased risk of T2D in females and males in Sweden.

Moreover, Gao et al. in a cross‐sectional study demonstrated that increased dietary intake of choline and betaine was associated with reduced insulin resistance [37]. The high heterogeneity among studies also reflects the existing controversy.

Choline‐associated diabetes might be influenced by the type and quantity of dietary fatty acids and suppliers of dietary choline/betaine, which may affect the ability of gut microbiota to synthesize TMAO. Although the main sources of dietary choline are red meat and eggs, consumption of other major dietary sources of this nutrient, such as poultry, soy foods, and fish, varies across the population [38, 39]. An inverse relationship between consumption of seafood and CVD incidence has been reported in several studies [40, 41]. Furthermore, omega‐3 fatty acids are believed to offer cardioprotective, antiarrhythmic, and antithrombotic effects [42]. Hence, adjusting for other dietary elements, including food suppliers of choline, energy intake, dietary cholesterol, polyunsaturated fatty acids, and saturated fatty acids, might present more reliable insight into the relationship between dietary choline and diabetes.

This is the case for the study conducted by Greenberg et al. [32], where adjusting for total dietary cholesterol removed the positive association of choline with diabetes risk. Nevertheless, some of the included studies did not adjust the above‐mentioned variables. Unfortunately, because of the limited number of studies considered, we could not conduct a subgroup analysis based on age, gender, study design, or any adjustment made for confounder variables. Hence, the significant heterogeneity can be linked to various factors. Differences in study design, such as methodologies, sample sizes, and participant demographics, play a crucial role in shaping outcomes. Moreover, individual biological variations, including genetic influences and gut microbiome composition, may also lead to differing health outcomes. Additionally, confounding elements such as lifestyle choices and pre‐existing health conditions can complicate the interpretation of results, resulting in varied findings across studies.

Betaine is involved in the conversion of homocysteine to methionine via its remethylation, a SAM (S‐adenosyl methionine) precursor. Betaine deficiency may reduce the synthesis of phosphatidylcholine, affect the synthesis and secretion of VLDL (very low‐density lipoprotein), and induce hepatic steatosis by reducing the availability of SAM [27]. It also may affect insulin signaling pathways by activation of IRS‐1(Insulin Receptor Substrate‐1), an initial step of insulin signaling, through its tyrosine phosphorylation. The role of betaine in glucose homeostasis by activation of protein kinase B (PKB/Akt) has also been reported [43]. Of the studies included in this meta‐analysis, only three assessed the relationship between dietary betaine and the risk of T2D, and none found significant results. Therefore, drawing a clear conclusion in this regard is difficult, highlighting the need for future well‐designed studies.

The result of the sensitivity analysis revealed that the study conducted by Virtanen et al. [8] had the largest effect on the results. After the exclusion of this study from the meta‐analysis, dietary choline intake showed a statistically significant positive relationship with diabetes risk. In the Virtanen et al. [8] study, dietary choline was assessed based on a 4‐day food record, whereas most of the included studies applied FFQ to evaluate the dietary intake of choline. Moreover, Virtanen′s study had a longer follow‐up duration and a lower number of total participants compared with the other studies, which may contribute to the significant heterogeneity in the findings of the sensitivity analysis.

The results of the ROBINS‐E assessment showed a moderate to serious risk of bias across the included studies, indicating concerns regarding confounding factors, misclassification, and exposure assessment. This intrinsic risk of bias can directly affect the reliability of the findings concerning dietary choline and betaine intake. Correspondingly, the results of the GRADE assessment revealed that the certainty of the evidence is very low for both choline and betaine intake regarding T2D incidence. This rating mirrors downgrades due to risk of bias, inconsistency, imprecision, and indirectness.

To improve the certainty of evidence, future studies should consider a comprehensive approach by conducting well‐designed randomized controlled trials and observational studies to guarantee comparability among various research efforts. Furthermore, including different populations in terms of age, gender, ethnicity, and health status can enhance the generalizability of findings. Comprehensive adjustments for possible confounders and performing longitudinal studies to assess the long‐term impacts of dietary choline and betaine, since dietary habits change over time, are also recommended. Finally, conducting subgroup analyses can help detect particular populations that may be more vulnerable to the impacts of dietary choline and betaine.

A thorough systematic literature search in online databases, the implementation of a detailed and strong approach to identify relevant studies, assessing the impact of an individual study on the overall result, and evaluating the certainty of evidence using the GRADE are some strengths of the current meta‐analysis. Conversely, the present study encountered several limitations compromising our findings. Since we performed a meta‐analysis of observational studies, unaccounted or residual confounders may influence the strength of the link between choline/betaine consumption and the risk of diabetes development. Although all the studies adjusted for possible confounding factors such as sex, age, BMI, smoking, and energy intake, some overlooked the consumption of other dietary elements such as the quantity and type of dietary fats, protein intake, a history of diabetes in the relevant family, primary food suppliers of betaine and choline, race, and physical activity. Moreover, inaccuracies in dietary assessment could impact the link between dietary choline/betaine and the risk of diabetes.

## 5. Conclusion

Our systematic review and meta‐analysis assessed the association of dietary choline/betaine and the risk of T2D based on observational studies. Our results did not indicate a significant link between dietary choline/betaine and the incidence of T2D. However, due to the limited number of studies, significant variability among them, and very low certainty of evidence, additional well‐designed, prospective research is needed to validate our findings.

## Disclosure

All authors reviewed the final version of the manuscript prior to submission.

## Conflicts of Interest

The authors declare no conflicts of interest.

## Author Contributions

E.S‐Z., S.M., N.N., and F.S.: participated in the research methodology and data gathering, interpretation, and writing of the manuscript. F.H‐S.: performed the statistical analysis and manuscript preparation. A.S.: contributed to the study design, thoroughly reviewed the paper, and authorized the submitted manuscript version.

## Funding

This study was supported by the Student Research Committee of Kermanshah University of Medical Sciences (no.4040749).

## Supporting Information

Additional supporting information can be found online in the Supporting Information section.

## Supporting information


**Supporting Information 1** Research Guideline Checklist: PRISMA checklist.

**Supporting Information 2** Table S1: Search strategy.

**Supporting Information 3** Table S2: Certainty assessment for the association between dietary choline and Type 2 diabetes. Table S3: Certainty assessment for the association between dietary betaine and Type 2 diabetes.

## Data Availability

Data were extracted from publicly available research articles. All datasets generated during this study can be available upon reasonable request from the corresponding author.
