# Supplementary material for: Dietary Choline and Betaine Are Not Associated With the Risk of Type 2 Diabetes. A Systematic Review and Meta‐Analysis of Observational Studies
Source: J Diabetes Res. 2026 Jan 1;2026:9980163. doi: 10.1155/jdr/9980163 (PMC12767234; doi:10.1155/jdr/9980163)
Supplement: Supplementary file 2 — Supporting Information 2 Table S1: Search strategy. [file JDR-2026-9980163-s002.docx]

Dietary Choline and Betaine Are not Associated With the Risk of Type 2 Diabetes. A Systematic Review and Meta-analysis of Observational Studies

Elham Sharifi-Zahabi et al

| ***Supplementary Table 1: Search Strategy*** | |
| --- | --- |
| *DUPLICATE =216*  *9/3/2024*  *SEP 2024* | ***Medline***  ((choline OR betaine OR Lecithin OR phosphatidylcholine OR phosphatidyl-choline) AND ( Diabetes OR diabetics OR diabetes mellitus) AND (observational study OR longitudinal studies OR prospective studies OR retrospective studies OR cohort studies OR population-based studies OR nested case-control studies OR nested case-control studies OR follow-up studies OR follow up studies OR Incidence Studies ))  N=419  ***Scopus***  *(( TITLE-ABS-KEY (choline) OR TITLE-ABS-KEY (betaine) OR TITLE-ABS-KEY (lecithin) OR TITLE-ABS-KEY (phosphatidylcholine) OR TITLE-ABS-KEY (phosphatidyl-choline)) AND (TITLE-ABS-KEY (*( Diabetes *) OR TITLE-ABS-KEY (* diabetics *) OR TITLE-ABS-KEY (* diabetes mellitus *) AND (TITLE-ABS-KEY (observational study) OR TITLE-ABS-KEY (longitudinal studies) OR TITLE-ABS-KEY (prospective studies)* *OR TITLE-ABS-KEY (retrospective studies) OR TITLE-ABS-KEY (cohort studies) OR TITLE-ABS-KEY (population-based studies) OR TITLE-ABS-KEY (population based studies) OR TITLE-ABS-KEY (nested case-control studies) OR TITLE-ABS-KEY (nested case-control studies) OR TITLE-ABS-KEY (follow-up studies) OR TITLE-ABS-KEY (follow up studies) OR TITLE-ABS-KEY (Incidence Studies))).*  *N=516*  ***Web of Science***  *(( ALL=(choline) OR ALL=(betaine) OR ALL=(lecithin) OR ALL=(phosphatidylcholine) OR ALL=(phosphatidyl-choline)) AND (ALL=(* Diabetes *) OR ALL=(* diabetics *) OR ALL= (* diabetes mellitus *) AND (ALL=(observational study) OR ALL=(longitudinal studies) OR ALL=(prospective studies)* *OR ALL=(retrospective studies) OR ALL=(cohort studies) OR ALL=(population-based studies) OR ALL=(population based studies) OR ALL=(nested case control studies) OR ALL=(nested case-control studies) OR ALL=(follow-up studies) OR ALL= (follow up studies) OR ALL=(Incidence Studies)))*  *N=518* |
