# Supplementary material for: Dietary Choline and Betaine Are Not Associated With the Risk of Type 2 Diabetes. A Systematic Review and Meta‐Analysis of Observational Studies
Source: J Diabetes Res. 2026 Jan 1;2026:9980163. doi: 10.1155/jdr/9980163 (PMC12767234; doi:10.1155/jdr/9980163)
Supplement: Supplementary file 3 — Supporting Information 3 Table S2: Certainty assessment for the association between dietary choline and Type 2 diabetes. Table S3: Certainty assessment for the association between dietary betaine and Type 2 diabetes. [file JDR-2026-9980163-s003.docx]

Dietary Choline and Betaine Are Not Associated With the Risk of Type 2 Diabetes. A Systematic Review and Meta-analysis of Observational Studies

**Author(s):** Sharifi-Zahabi et al.

**Supplementary Table 2: Certainty assessment for the association between dietary choline and type 2 diabetes.**

| **Certainty assessment** | | | | | | | **№ of patients** | | **Effect** | | **Certainty** | **Importance** |
| --- | --- | --- | --- | --- | --- | --- | --- | --- | --- | --- | --- | --- |
| **№ of studies** | **Study design** | **Risk of bias** | **Inconsistency** | **Indirectness** | **Imprecision** | **Other considerations** | **High intake of choline** | **Low intake of choline** | **Relative (95% CI)** | **Absolute (95% CI)** |  |  |
| **Diabetes mellitus** | | | | | | | | | | | | |
| 5 | non-randomised studies | very serious^a^ | very serious^b^ | very serious^c^ | serious^d^ | none | 2106/14710 (14.3%) | 1562/14666 (10.7%) | **OR 1.15** (1.00 to 1.33) | **14 more per 1,000** (from 0 fewer to 30 more) | ⨁◯◯◯ Very low^a,b,c,d^ | CRITICAL |

**CI:** confidence interval; **OR:** odds ratio

#### Explanations

a. More than 20% of studies for this outcome had a high risk of bias for at least one component of the risk of bias tool. Those biases had a significant effect on the results of studies.

b. Heterogeneity among the studies was high (I2>50%) and results were from moderate-to-low-quality evidence.

c. Cut-off points used to determine the unexposed group varied widely across studies, with the range of the unexposed group in one study being the range of the exposed group in another.

d. 95% CI include the non-significant value.

Suplementary Table 3:**Certainty assessment for the association between dietary betaine and type 2 diabetes.**

| **Certainty assessment** | | | | | | | **№ of patients** | | **Effect** | | **Certainty** | **Importance** |
| --- | --- | --- | --- | --- | --- | --- | --- | --- | --- | --- | --- | --- |
| **№ of studies** | **Study design** | **Risk of bias** | **Inconsistency** | **Indirectness** | **Imprecision** | **Other considerations** | **High intake of betaine** | **Low intake of betaine** | **Relative (95% CI)** | **Absolute (95% CI)** |  |  |
| **Diabetes mellitus** | | | | | | | | | | | | |
| 3 | non-randomised studies | very serious^a^ | very serious^b^ | very serious^c^ | very serious^d^ | none | 1272/11252 (11.3%) | 989/9210 (10.7%) | **OR 0.99** (0.90 to 1.10) | **1 fewer per 1,000** (from 10 fewer to 9 more) | ⨁◯◯◯ Very low^a,b,c,d^ | CRITICAL |

**CI:** confidence interval; **OR:** odds ratio

#### Explanations

a. More than 20% of studies for this outcome had a high risk of bias for at least one component of the risk of bias tool. Those biases had a significant effect on the results of studies.

b. Heterogeneity among the studies was high (I2>50%) and results were from moderate-to-low-quality evidence.

c. Cut-off points used to determine the unexposed group varied widely across studies, with the range of the unexposed group in one study being the range of the exposed group in another.

d. 95% CI include the non-significant value.
